# Wave-shaped temperature dependence characteristics of the electroluminescence peak energy in a green InGaN-based LED grown on silicon substrate

**DOI:** 10.1038/s41598-019-57008-3

**Published:** 2020-01-10

**Authors:** Changfu Li, Jianfei Li, Mingsheng Xu, Ziwu Ji, Kaiju Shi, Hongbin Li, Yehui Wei, Xiangang Xu

**Affiliations:** 1 School of Microelectronics, Shandong University, Jinan, 250100 China; 2 School of Physics and Electronic Engineering, Taishan University, Taian, 271000 China; 3 School of Science, Qilu University of Technology, Jinan, 250353 China; 4 State Key Laboratory of Crystal Materials, Shandong University, Jinan, 250100 China

**Keywords:** Inorganic LEDs, Photonic devices

## Abstract

This study aimed to investigate temperature dependencies at different injection currents (ICs) of the electroluminescence (EL) spectra from a green InGaN/GaN light-emitting diode (LED) based on multiple quantum wells (MQWs) grown on a Si substrate in a wide range of ICs (0.001–350 mA) and temperatures (6–350 K). The results show that the temperature-changing characteristic of the EL peak energy gradually evolves from an approximately V-shaped temperature dependence into a wave-shaped (three-step blueshift) dependence with increasing IC. Finally, it emerges as an approximately inverted V-shaped temperature dependence. The behavior reflects the fact that the emission related to InGaN is significantly influenced by the changing recombination dynamics of carriers with rising temperature or IC. This is attributed to the presence in the MQW active region of a stronger carrier localization effect across three zones with different average In contents. Moreover, with the decline of the temperature at lower ICs, the temperature behavior of the external quantum efficiency (EQE) value is dominated by the deactivated non-radiative centers. This phenomenon occurs not only in the higher temperature range but also at lower temperatures due to more In-content-induced structural defects, which are confirmed by measurements of the integrated EL intensity as well as the EQE dependence on IC.

## Introduction

Light-emitting diodes (LEDs) based on InGaN/GaN multiple quantum wells (MQWs) have attracted considerable attention and entered the market for solid state lighting as the emitted light from UV through the visible spectral range is tenable^1–4^. In recent years, blue InGaN/GaN MQWs-based LEDs have an external quantum efficiency (EQE) of 84.3%^5^. In contrast, InGaN/GaN MQWs-based LEDs which emit light of longer wavelength (in the green/yellow spectral range), usually suffer from a reduction of emission efficiency (that is, the alleged green gap). These characteristics are owing to the fact that high In-content-induced composition fluctuation, or phase separation, results in the occurrence of the structural defects which serve as a non-radiative recombination center in the InGaN epilayers^6–8^. Therefore, understanding the influence mechanisms of the fluctuating components, or phase separation on the recombination of carriers in the InGaN-related materials is of significant importance towards improving the performance of these optics devices.

In high In-content InGaN/GaN MQW structures, the radiative recombination is essentially due to the localization of carriers within In-rich regions resulting from composition fluctuations or partial phase segregation in the well layers of InGaN. Many different optical characteristics including the increasing temperature-induced thermal expansion and relaxation of localized carriers^9,10^, the band-filling for localization states of injected carriers^11,12^, and the enhancement of carrier transfer from shallow to deep localization states induced by carrier-scattering effect^13^, can be elucidated. Moreover, an inverted V-shaped temperature-dependent characteristic of the emission peak energy in a wide fixed injection current range is attributed to the higher homogeneity at the depths of these localized states^14^. Despite these, the carrier dynamics of the anomalous behaviors induced by temperature, especially for the EL in the InGaN/GaN MQWs-based LEDs with high In-contents, remain to be fully determined.

In this study, to determine the potential physics of light emission from InGaN/GaN MQWs-based LED emitting photons at green wavelengths, the temperature dependencies at varying ICs of electroluminescence (EL) spectra were measured and investigated by analyzing the EL peak energy, peak linewidth and EQE.

## Results and Discussion

**Figure 1:**
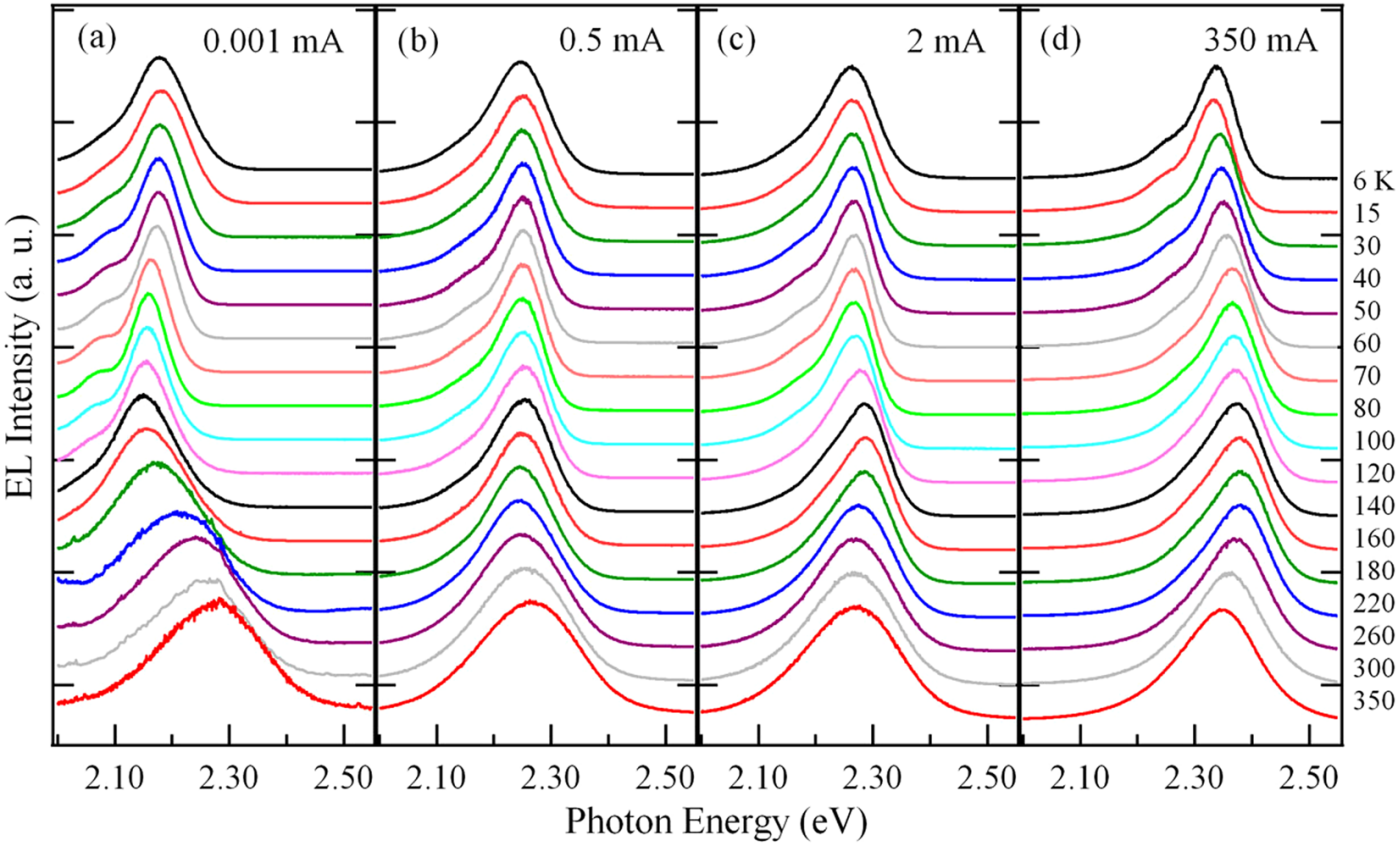
Temperature-dependent EL spectra of the green InGaN/GaN MQWs-based LED measured at 0.001 (**a**), 0.5 (**b**), 2 (**c**), and 350 mA. (**d**) The weak peaks on the low energy side of the main EL peak are phonon replicas of the main peak.

**Figure 2:**
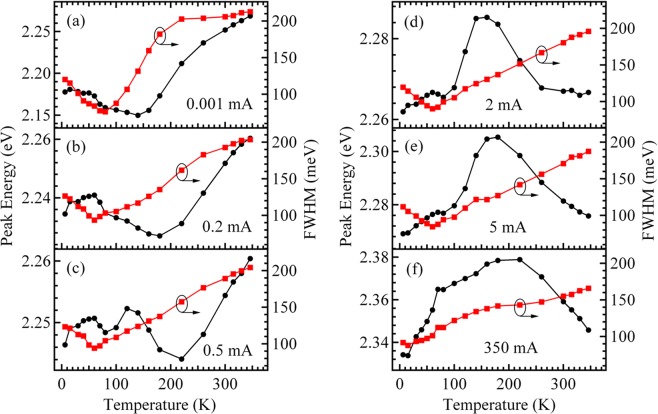
Temperature dependencies of the EL peak energy and full width at half maximum (FWHM) for the green LED measured at 0.001 (**a**), 0.2 (**b**), 0.5 (**c**), 2 (**d**), 5 (**e**), and 350 mA (**f**).

Figure 1 shows the temperature-dependent EL spectra of the green InGaN/GaN MQWs-based LED measured from 6 to 350 K at the fixed ICs of 0.001, 0.5, 2, and 350 mA. Analysis of the data shows that each spectrum is dominated by one asymmetric main EL peak with a low energy tail, originating from MQWs of the green LED. The weak peaks on the low energy side of the main EL peak are phonon replicas of the main peak. The representative temperature dependencies of the main EL peak energy and linewidth for emissions at fixed currents of 0.001, 0.2, 0.5, 2, 5, and 350 mA are displayed in Fig. 2. It can be seen from Fig. 2 that the EL peak energy shows an anomalous temperature-dependent behavior.

**Figure 3:**
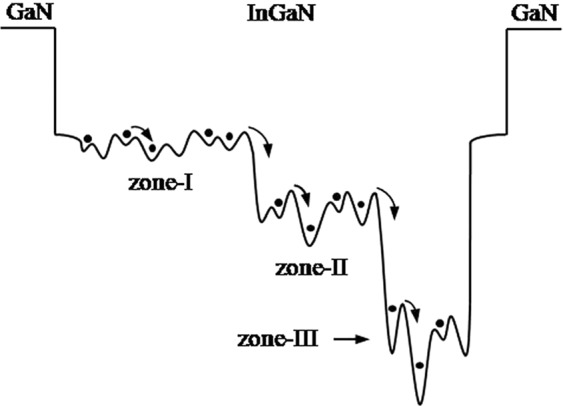
A sketch map for the transfer and distribution of carriers in the MQWs containing three zones with different In contents.

To explain the anomalous temperature-related behaviors of the EL peak energy at different ICs in Fig. 2, Fig. 3 demonstrates the underlying mechanism for the transfer and distribution of carriers in the MQW structure. It can be seen from Fig. 3 that there are three zones with different average values of In content in the green MQW structure: an In-poor zone (marked as zone-I) with a very slight compositional fluctuation and smaller localization energy, an In-intermediate zone (zone-II) with moderate compositional fluctuation and moderate localization energy, and an In-rich zone (zone-III) with significant compositional fluctuation and larger localization energy. The emissions relating to zones-II and -III should be at the lower energy side (low energy tail) of the asymmetric main EL peaks as shown in Fig. 1.

From Fig. 3, it can be seen that at the lowest temperature (6 K) and lowermost fixed current (0.001 mA), carriers exhibit random distribution among the potential minima in the MQWs (*i*.*e*. inside these three zones)^15,16^. However, when the temperature increases to 350 K from 6 at 0.001 mA, Fig. 2(a) shows that the temperature behavior of the peak energy presents an approximate “V shape”: the peak energy decreases slightly as the temperature ranges from 6 to 60 K, decreases markedly from 60 to 70 K, decreases from 70 to 140 K, and increases significantly from 140 to 350 K. At the same time, the linewidth decreases below the critical temperature of about 80 K, increases significantly from 80 to 220 K, remains almost constant from 220 to 300 K, and increases slightly from 300 to 350 K with increasing temperature. These data provide the following hints: as the temperature rises from 6 to 350 K, first, the localized carriers relax from shallower down into deeper localization states inside their respective zones and from the shallower localized zone-I down into the deeper localized zone-II followed by zone-III below about 140 K; then, the thermal broadening effect of the localized carriers in the MQWs including these three zones begins to dominate the emission process with further increasing temperature above 140 K^13,17–21^.

With increasing fixed current to 0.5 mA from 0.001, it can be seen from Fig. 2(a–c) that, differing from the case at 0.001 mA, at 0.2 mA, the peak energy slightly increases with increasing temperature from 6 to 60 K; further, differing from the case at 0.2 mA, at 0.5 mA, the peak energy increases first and then decreases (*i*.*e*. an energy peak occurring) with increasing temperature from 80 to 220 K. That is, at 0.5 mA, the peak energy displays a wave-shaped (three-step blueshift) temperature behavior: the peak energy increases with a change at temperatures from 6 to 60 K, then decreases slightly from 60 to 80 K. This is followed by a slight increase from 80 to 120 K, a decrease from 120 to 220 K and then a significant increase from 220 to 350 K. At the same time, the linewidth also displays V-shaped temperature behavior at 0.5 mA, similar to that observed at the case of 0.001 mA in Fig. 2(a), however, the former shows a lower critical temperature (60 K) than the latter (80 K). These behaviors can be explained by the fact that the relaxation process of the localized carriers related to the temperature inside the respective zones is gradually suppressed with increase of the fixed current from 0.001 to 0.5 mA. This may be due to the gradual reduction of the carrier localization effect inside their respective zones ^14,15^. In contrast, the relaxation process of the localized carriers dependent on temperature from the shallower downwards to the deeper localized zone structure is still in progress due to the larger difference in the potential minima between these three zones, originating from their larger difference in the average In content. Therefore, as the temperature increases to 350 K from 6 at 0.5 mA, the dominant temperature behavior of the peak energy is the thermalization of the carriers which are subjected to localization inside the zone-I from 6 to 60 K, followed by relaxation of the localized carriers from the zone-I down into zone-II or by the relative enhancement of emissions from the zone-II from 60 to 80 K owing to its stronger localization effect than the zone-I. This is followed by thermalization of localized carriers inside zone-II from 80 to 120 K, and subsequently by relaxation of localized carriers from the zone-II down into the zone-III or by the relative enhancement of the emissions from the zone-III from 120 to 220 K due to its stronger localization effect than the zone-II. Finally, the thermalization of localized carriers in the MQWs (*i*.*e*. inside these three zones) from 220 to 350 K occurs ^13,17,18,22,23^.

Here, it is worth noting that, the aforementioned wave-shaped (three-step blueshift) temperature behavior of the peak energy at 0.5 mA (Fig. 2(c)), cannot be explained by the temperature-dependent recombination mechanisms of the localized carriers either in the blue InGaN/GaN MQWs including only one zone with a slight compositional fluctuation or in the green InAlGaN thin film including two zones with a smaller compositional fluctuation and a relatively larger compositional fluctuation. This is because the former exhibits only a single S-shaped (redshift-blueshift-redshift) temperature dependence of the peak energy (i.e. one-step blueshift), and the one-step blueshift is attributed to the thermal broadening effect of the localized carriers in the MQWs including only one zone^10,14,15^; the latter exhibits only a double S-shaped (redshift-blueshift-redshift-blueshift-redshift) temperature dependence of the peak energy (i.e. two-step blueshift), and the two-step blueshift is attributed to the thermal broadening effect of the localized carriers in the two zones with different potential barriers^24^.

When the fixed current increases to 350 mA from 0.5 (as can be seen from Fig. 2(c–f)), the temperature-changing characteristic of the EL peak energy gradually evolves from the wave-shaped temperature dependence into an approximately inverted V-shaped temperature behavior: the peak energy at 350 mA increases at a higher rate in the range of 6–70 K, then grows at a lower rate in the range of 70–220 K, finally decreases closely following Varshni’s law with increasing temperature in the range of 220–350 K. At the same time, the linewidth has a monotonically increased temperature behavior at 350 mA. These behaviors are due to the fact that when the fixed current increases to 350 mA from 0.5, the temperature-dependent relaxation process of the localized carriers between these three zones is also gradually suppressed due to a further declining localization effect of carriers in the MQWs (*i*.*e*. inside these three zones) at the highest current of 350 mA. Therefore, at 350 mA, the temperature behavior of peak energy is dominated first by thermalization of localized carriers inside these three zones from 6 to 220 K. Then, the dominated temperature behavior is shown as the regular thermalization of free carriers in the MQWs for the highest full-delocalization temperature range of 220–350 K^14,15,25^.

**Figure 4:**
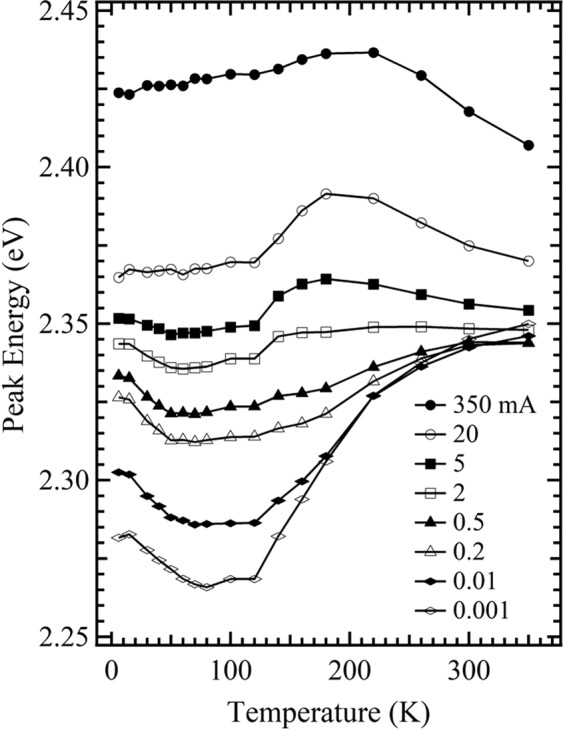
Temperature dependencies of the EL peak energy for the sub-green LED measured at 0.001, 0.01, 0.2, 0.5, 2, 5, 20, and 350 mA.

In addition, it should be pointed out that as a sister sample of the green LED used in this study, another green LED (denoted as a sub-green LED) with a shorter emission wavelength due to its higher growth temperature compared with the green LED^13^, shows a different temperature behavior from that observed in Fig. 2 for the green LED: when the IC increases to 350 mA (from 0.001 mA or less), the dependence of the EL peak energy on temperature in the range of 6–350 K, displays a gradual change from a “markedly decrease-remaining almost constant-significantly increase” temperature dependence to a V-shaped (slightly decrease-slowly increase) relationship, followed by a “slightly decrease-slightly increase-remaining almost constant” and a “remaining almost constant-increase-decrease” one, till the dependence shows an inverted “V shape” (slowly increase-markedly decrease) (Fig. 4). That is, the Wave-shaped (three-step blueshift) temperature dependence of the EL peak energy for the green LED, as shown in Fig. 2, was not observed over the whole measured range in Fig. 4 for the sub-green LED. This phenomenon seems to indicate that there may be two or three zones with smaller difference in the average values of In content in InGaN well layers for the sub-green LED compared to the green LED. Also, in previous reports^14,15^, we used a blue LED with the same chip size and assembled InGaN/GaN MQW structure, but disparate substrates (sapphire) in comparison to that when the green LED is used. The previously described temperature behavior of the EL peak energy for the green LED shown in Fig. 2, is also not observed across the whole of the measured range for the blue LED. As the IC (excitation power) increases to 200 mA (50 mW) from 1 µA or less (0.5 µW or less), the dependence of the EL (photoluminescence, PL) peak energy on temperature in a range of 6–300 K for the blue LED shows a gradual evolution from a S-shaped (decrease-increase-decrease) form, to an inverted “V shape”. This indicates that, the blue counterpart has a weaker localization effect due to the slight fluctuation of components in the InGaN well layer compared to the green LEDs.

**Figure 5:**
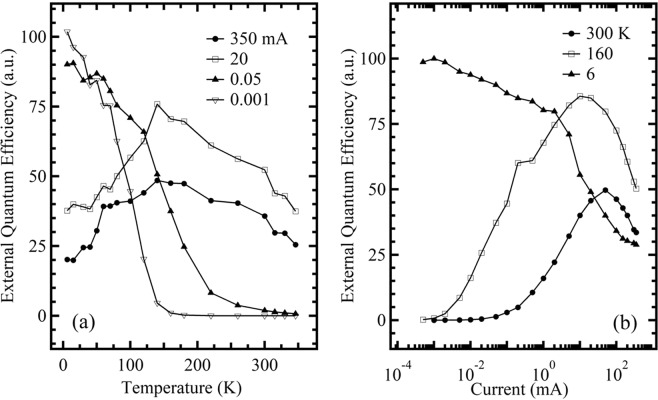
Dependencies of relative EQE for the green LED on temperature at the fixed currents of 0.001, 0.05, 20, and 350 mA (**a**), and on current at the fixed temperatures of 6, 160, and 350 K (**b**).

To investigate the transfer and recombination mechanisms of the carriers in the green LED structure further, we plotted Fig. 5(a), in which the integrated EL intensity subjected to division by the current (that is, relative EQE) is shown as a function of temperature at typical fixed currents of 0.001, 0.05, 20, and 350 mA. From Fig. 5(a), it can be seen that when the temperature declines from 350 to 6 K, at lower fixed currents below 5 mA, the value of EQE displays a monotonous increase, in contrast, at higher fixed currents above 5 mA, the value of EQE exhibits an increase and then a decrease. The latter is similar to those of the aforementioned blue LED observed in the entire measurement range in our previous works^14,26^, and may be explained by the fact that, as the temperature declines from 350 to 6 K, the non-radiative recombination centers are gradually deactivated first, and then electron leakage gradually rises with further decreasing temperature due to the augment of the forward bias; however, the former shows that the deactivation process of the non-radiative centers not only occurs in the range of higher temperature but also in the range of lower temperature. These behaviors may be attributed to the fact that the green LED has more defect-related non-radiative centers than the blue counterpart due to the high In content, because the measurement results of the dependence of the integrated EL intensity on IC in the lower IC range and at 300 K for green and blue LEDs, have shown that the former has a larger *F* value (2.531) than the latter (1.749) (not shown)^26^. Here, it should be noted that in addition to the defect-related non-radiative centers, the composition fluctuation results in generation of the In-rich regions, which act as carrier localization centers, to enhance quantum efficiency. Therefore, the higher EQE at lower temperatures and at lower fixed currents (Fig. 5(a)) is also related to the stronger carrier localization effect, in addition to smaller electron leakage under these conditions. The injection current-dependent reduction of the carrier localization effect induced by composition fluctuation can be attributed to the fact that, when the IC gradually increases, corresponding to the gradual increase of the external electric field, in addition to the localization centers being gradually filled by the increasing injection carrier, the QW profile gradually inclines, resulting in the carrier easily escaping from the localization centers^14,26,27^.

Moreover, the EQE is also plotted as a function of injection current at the fixed temperatures of 6, 160, and 350 K in Fig. 5(b). It is found from Fig. 5(b) that, when the current increases from 0.5 µA to 350 mA, the EQE value monotonically decreases at the lower fixed temperature of 6 K, in contrast, the EQE value markedly increases first and then monotonically decreases at the higher fixed temperatures, such as at 160 and 300 K. The former is mainly attributed to the marked electron leakage over the entire current range^26,28^; the latter is mainly due to the recombination mechanism conversion from non-radiative to radiative recombination in the lower current range, and to the marked electron leakage and/or overflow in the higher current range^26,28^. The explanations are also in good agreement with that with respect to Fig. 5(a).

## Conclusions

This study investigated the EL spectra of a green InGaN/GaN MQWs-based LED grown on a Si substrate in wide IC (0.001–350 mA) and temperature (6–350 K) ranges. It was found that, as the fixed current increases from 0.001 to 350 mA, the dependence of the peak energy on temperature displays a gradual evolution from an approximately V-shaped temperature dependence to a wave-shaped (three-step blueshift) relationship, until the dependence is changed to an approximately inverted “V shape”. The behavior reflects the fact that the emission related to InGaN is significantly influenced by the changing recombination dynamics of carriers with increasing temperature or IC. This can be attributed to the presence in the MQW active region of the stronger carrier localization effect. Also, three zones with different degrees of component fluctuation and carrier localization effects are due to their different average values of In content. Moreover, at lower fixed currents and temperatures, the dependence of the EQE value on temperature is dominated by deactivated non-radiative centers, even in the lower temperature range. This is attributed to the more numerous high-In-content-induced structural defects in the green MQW structure, which is in agreement with the results obtained from tests of the dependence of the integrated EL intensity as well as the EQE upon IC. The experimental results provide further support for the development of high-performance LEDs emitting photons at long wavelengths.

## Methods

### Sample fabrication

The green InGaN/GaN MQWs-based LED was grown on a Si substrate oriented to (111) through the metalorganic chemical vapor deposition (MOCVD). The LED structure comprised an AlN low-temperature nucleation layer, an AlN/AlGaN insertion layer, an unintentionally doped GaN buffer layer, a n-GaN layer doped with Si, six pairs of InGaN/GaN MQWs (as active region) with 2 nm thick InGaN wells and 14 nm thick GaN barriers, and a p-AlGaN electron blocking layer (EBL). This was followed by a p-GaN contact layer doped with Mg. The In content in the QWs was estimated to be 32%. Details of the growth procedure are reported elsewhere^13^, with the exception of its lower growth temperature. The LED chip measured 1.16 × 1.16 mm around was produced by adopting the conventional mesa-structure method.

### Measurements

Temperature and current-related EL were measured by installing a chip on a Cu cold-stage in a He cryostat with a closed cycle where the temperature changed in the range of 6–350 K. To reduce self-heating effect, the EL was excited by a pulsed current with a pulse width of 7 ms and a duty cycle of 20%, which was produced by applying a constant-current 2400 Source Meter (Keithley, the United States) equipped with a MOS switch. The pulsed current was changed in the range of 0.001–350 mA. Signals from the sample were dispersed and detected by separately using a Jobin-Yvon iHR320 monochromator and a thermo-electrically cooled Synapse CCD detector.
